# Recent Advances in the Study of the Immune Escape Mechanism of SFTSV and Its Therapeutic Agents

**DOI:** 10.3390/v15040940

**Published:** 2023-04-10

**Authors:** Lei Chen, Tingting Chen, Ruidong Li, Yingshu Xu, Yongai Xiong

**Affiliations:** 1 Key Laboratory of Basic Pharmacology of Guizhou Province and School of Pharmacy, Zunyi Medical University, Zunyi 563000, China; 2 Key Laboratory of Basic Pharmacology of Ministry of Education and Joint International Research Laboratory of Ethnomedicine of Ministry of Education, Zunyi Medical University, Zunyi 563000, China

**Keywords:** SFTS, pattern recognition receptors, immune escape, mechanism, therapeutic agents

## Abstract

Sever fever with thrombocytopenia syndrome (SFTS) is a new infectious disease that has emerged in recent years and is widely distributed, highly contagious, and lethal, with a mortality rate of up to 30%, especially in people with immune system deficiencies and elderly patients. SFTS is an insidious, negative-stranded RNA virus that has a major public health impact worldwide. The development of a vaccine and the hunt for potent therapeutic drugs are crucial to the prevention and treatment of Bunyavirus infection because there is no particular treatment for SFTS. In this respect, investigating the mechanics of SFTS–host cell interactions is crucial for creating antiviral medications. In the present paper, we summarized the mechanism of interaction between SFTS and pattern recognition receptors, endogenous antiviral factors, inflammatory factors, and immune cells. Furthermore, we summarized the current therapeutic drugs used for SFTS treatment, aiming to provide a theoretical basis for the development of targets and drugs against SFTS.

## 1. Introduction

In 2009, The National Center for Disease Control and Prevention of China received test samples from a patient in Hubei Province, China, who had identical signs to a patient from Hubei province with symptoms of fever, malaise, leukopenia, and thrombocytopenia. The Chinese Center for Health CDC then isolated a novel virus from this patient, and it was later identified as SFTSV1 after being found in all 81 identical patients who presented in the ensuing months [1]. Since then, reports of the virus’s emergence have come from Japan [2], South Korea [3], Pakistan [4], Vietnam [5], and many other nations and areas. One of the primary vectors for this virus is ticks, and infected ticks that attach to humans and draw blood from them release and transmit SFTSV as a result of this blood-sucking process [6,7]. As a result, people who have had high levels of interaction with ticks are more likely to develop fever with thrombocytopenia syndrome. Additionally, recent research indicates that cats are extremely susceptible to SFTSV, so it is important to take into account the possibility of human infection in cats with SFTS [8]. The incubation period following SFTSV infection is typically 5 to 14 days. Fever, gastrointestinal discomfort, and thrombocytopenia, and in extreme instances, pancreatic injury, myocardial injury, and even central nervous system lesions and encephalitis, are common clinical manifestations [1,9,10]. In this overview, the virology of the virus, interactions between the virus and its hosts, and antiviral drug research are all summarized and discussed.

Bunyaviruses are the largest known family of RNA viruses, and more than 350 viruses have been identified, classified according to serological, morphological, and biochemical characteristics into the genera Orthobunyavirus, Nairovirus, Phlebovirus, Hantavirus, and Tospovirus, as well as other unclassified viruses [11]. In 2011, Yu et al. identified SFTSV as a spherical, enveloped, and segmented negative-stranded RNA virus belonging to the genus Phlebovirus Bunyavirus by whole genome determination of 12 SFTSV strains isolated from Chinese SFTS patients. The genome of this viral genus consists of a group of segmented single-stranded negative-sense RNA motifs encoding small (S), medium (M), and large (L) segments [1], which are structurally similar to those of other bunyaviruses [12].

SFTSV is highly similar in genetic structure to Heartland virus and Guertu virus of the same genus [13,14]. The HV virus was isolated and discovered in Missouri, USA, in 2012, which also belongs to the genus Phlebovirus [15], and Guertu virus was discovered in 2014 from Xinjiang province, China [14]. Encoding viral nucleocapsid proteins (NPs) and nonstructural proteins (NSs) were identified on the S fragment of SFTSV, the most abundant protein in SFTSV particles and infected cells, which wraps the RNA (vRNA) of the viral genome and leads to the formation of ribonucleoprotein complexes (RNPs). NPs play a protective role in viral replication and assembly and are responsible for encapsulating and protecting viral genomic RNA from degradation by exogenous nucleases and the innate immune system in the host cell [16]; NSs are also important virulence protein factors of SFTSV and are strong inhibitors of IFN production, which inhibit the natural antiviral response of the host cell in several ways [17].

It has also been demonstrated that NS proteins can form viral plasma-like structures (VLSs) in infected and transfected cells, and viral dsRNA is localized in the VLS of infected cells, suggesting that VLSs formed by NSs may be associated with SFTS bunyavirus replication [18]. Numerous studies have reported that NSs and NPs inhibit host cell antiviral immune responses by suppressing interferon and cytosolic nuclear factor signaling, and lead to multi-organ dysfunction. In addition, the S segment encodes a nuclear protein of SFTSV, which inhibits the activation of IFN-β and NF-κB signaling pathways [19]. Besides, the M segment encodes a nuclear protein precursor (Gp), which is cleaved and modified by proteases during translation to yield structural glycoprotein N (Gn), glycoprotein C (Gc) monomers, and Gn/Gc proteins, which mediate viral entry into host cells via pH-dependent endocytosis, while Gn promotes early SFTSV infection, interacts with host cell receptors, and serves as a major target for neutralizing immune responses. The L fragment specifies the viral gene’s RNA-dependent RNA polymerase (RdRp), which is responsible for viral RNA replication and mRNA synthesis [20].

Furthermore, it has been demonstrated that the L segment is capable of initiating viral RNA replication and mRNA synthesis without the use of primers or reassignment. The L segment most likely starts genome replication on vRNA without the use of primer and reassignment mechanisms [21]. Recombination and partial reassignment on SFTSV S, M, and L sequences may also enable the virus to evolve and evade the host immune response. Venous viral serocomplexes are a class of serum cross-reactive viruses that are phylogenetically related to and transmitted by arthropod vectors. This group of viruses can also be spread by mosquitos (e.g., Rift Valley fever virus; RVFV) or sand flies, in addition to ticks (e.g., Tuscany virus).

## 2. Viral Evasion of Immunization Modes

SFTSV entry into the host cell is initiated by recruitment of lattice proteins to the cell membrane to form lattice-protein-coated pits and further extrusion from the plasma membrane to form discrete vesicles. These vesicle carriers further deliver viral particles to Rab5+ early endonucleosomes and then to Rab7+ late endonucleosomes. Intracellular transport of endocytic vesicles carrying viral particles is dependent first on actin filaments at the cell periphery and then on microtubules inside the cell. The final fusion event occurs approximately 15–60 min after entry and is triggered by an acidic environment of ≈pH 5.6 within the late nucleosome. In addition, SFTSV entry into target cells requires Gn/Gc binding to cellular receptors and fusion of the virus with the cell membrane, a process catalyzed by conformational changes in viral proteins and subsequent release of viral RNA into the cytoplasm [22].

## 3. Innate Immunity

The innate immune response is the first line of defense against SFTSV infection; moreover, the ability of the host to suppress viral infection relies heavily on the effectiveness of the initial antiviral innate immune response, and activation of innate immune cells is critical to initiate adaptive immunity. Innate immune cells use pattern recognition receptors (PRRs) to recognize pathogen-associated molecular patterns (PAMPs) and danger-associated molecular patterns (dAMPs) to detect viruses [23]. Upon entry into the body, SFTSV dsRNA is recognized and signaled to the next junction by pattern recognition receptors such as RIG-like receptors and Toll-like receptors.

IFN: Interferons are powerful antiviral cytokines that induce various antiviral responses to inhibit viral replication. Interferons are divided into three types (type I, type II, and type III) based on sequence homology, with type I and type III IFNs being more important. Type I interferons are further divided into thirteen subtypes including IFN-α and IFN-β, and type III IFNs are divided into IFN-λ1, IFN-λ2, and IFN-λ3 [24]. In the recognition process of SFTSV-infected cells, RIG-I is the main recognition receptor for the viral RNA sensor molecule, which makes the IFN-β promoter stimulator 1 (IPS-1) or the mitochondrial antiviral signaling protein MAVS. IPS-1 signals to TANK-binding kinase 1 (TBK1) and NF-κB kinase inhibitor (IKK) to induce type I IFN secretion by activating the phosphorylation of IFN regulators IRF3 and IRF7 [25].

TANK-binding kinase 1 (TBK1): TBK1 (tumor necrosis factor (TNF) receptor-associated factor NF-κB activator (TANK)-binding kinase 1), is involved in the activation of multiple cellular pathways leading to the production of IFN and proinflammatory cytokines after infection, autophagic degradation of protein aggregates or pathogens, and homeostatic cellular functions such as cell growth and proliferation [26]. During SFTSV invasion of host cells, the interaction of NSs with TBK1 is an important pathway for inhibition of host interferon, and NSs bind to TBK1 to isolate it into NS-induced cytoplasmic structures, a critical step in SFSTV evasion of host antiviral responses. SFTSV NSs associate with TBK1 through its N-terminal kinase structural domain, thereby blocking the autophosphorylation of TBK1, which ultimately inhibits IFN production [27]. Moreover, in a report, it was demonstrated that SFTSV can inhibit the induction of IFN-a and IFN-b through NS–IRF7 interactions and the isolation of IRF7 in the viral envelope [28].

STAT2 (signal transducer and activator of transcription) is an important factor in IFN production. Phosphorylated homodimers or heterodimers of STAT2 together with IRF9 form the ISGF3 complex, which translocates into the nucleus, binds to specific IFN stimulatory responses in the promoters of IFN-stimulated gene (ISG) elements (ISREs) in the promoter of IFN-stimulated genes (ISGs), and activates ISGs’ transcription [29]. During the IFN signaling phase, SFTSV NS proteins inhibit IFN signaling and ISGs’ expression by segregating STAT2 into inclusion bodies (IBs) and impairing STAT2 heterodimer phosphorylation and nuclear translocation [30]. Furthermore, in a study, Xu Chen et al. first found that SFTSV inhibits exogenous IFN α-induced Jak/STAT signaling by reducing Y701 p-STAT1 levels, inhibiting IFN stimulatory response element ISRE activity, and downregulating ISGs’ expression [31]. Both studies suggest that SFTSV NS proteins can inhibit exogenous IFN α-induced Jak/STAT signaling by suppressing STAT1 phosphorylation and activation, and that SFTSV inhibits Jak/STAT signaling to inhibit type I and type III IFN signaling. In addition to this, in an in vivo experiment in mouse animals, SFTSV was found to induce fatal acute disease in STAT2-deficient mice, but not in STAT1-deficient mice. This is due to the inability of NSs to bind to mouse STAT2 and inhibit type I IFN signaling in mouse cells. This implies that dysfunction of NSs in antagonizing mouse STAT2 can lead to inefficient replication and loss of pathogenesis of SFTSV in mice [32]. This was likewise argued in another animal experimental model, where Gowen et al. demonstrated that hamsters lacking functional STAT2 were highly sensitive to SFTSV down to 10 PFU and that animals usually died within 5 to 6 days after subcutaneous stimulation [33].

TRIM21 and TRIM25. Most of the TRIM family proteins have E3 ubiquitin ligase activity and play multiple functions in intracellular signaling, development, apoptosis, protein amount control, innate immunity, autophagy, and carcinogenesis. There is growing evidence that TRIM family proteins have unique and important roles, and their dysregulation leads to several diseases such as cancer, immune disorders, or developmental disorders [34]. TRIM21 mediates monoubiquitination and subcellular translocation of active IKKB to autophagosomes and inhibits IKKB-mediated NF-κB signaling [35]. Choi, Y. et al. demonstrated that the SFTSV NSs inhibits the TRIM21 function to upregulate the p62-Keap1-Nrf2 antioxidant pathway for efficient viral pathogenesis [36]. Besides, TRIM25-mediated k63-linked ubiquitination of retinoic acid-inducible gene I (RIG-I), which induces RIG-I-mediated IFN production [37], revealed that SFTSV NSs can specifically capture TRIM25 into viral inclusion bodies and inhibit TRIM25-mediated ubiquitination/activation of the RIG-Lys-63 linkage, contributing to the inhibition of RLR-mediated antiviral signaling during its initial phase [38].

IRF family. Interferon regulatory factors (IRFs) are a family of homologous proteins responsible for the regulation of IFN transcription and IFN-induced gene expression. They are important regulatory proteins in the Toll-like receptor (TLR) and IFN signaling pathways, which are essential elements of the innate immune system [39]. Studies have demonstrated that, during SFTSV invasion of host cells, its NSs chelate TBK1 into the cytoplasmic structure induced by NSs, indirectly inhibiting IRF3 and subsequent IFN-β production. It has also been demonstrated that NSs with 21 and 2 conserved amino acids at position 23 are essential for the inhibition of IRF3 phosphorylation and IFN-β mRNA expression [40]. Ye Hong et al. demonstrated that NSs directly interact with IRF7 and chelate it into the inclusion bodies, unlike IRF3, which indirectly interacts with NSs. Although the interaction of NSs with IRF7 did not inhibit IRF7 phosphorylation, p-IRF7 was trapped in the inclusion bodies, leading to a significant reduction in IFN-2, IFN4 induction, and thus enhanced viral replication [28]. In addition, LSm14A, a member of the LSm family, is involved in RNA processing in the processing body, binds to viral RNA or synthetic homologs, and mediates IRF3 activation and IFN-β induction, and it has been shown that the cellular NS proteins interact and co-localize with LSm14A, and this interaction effectively inhibits downstream phosphorylation and dimerization of IRF3, thereby inhibiting antiviral signaling pathways and IFN induction in several human cell types. The SFTSV NS LRRD motif binds to the LSm14A–NS protein complex, thereby affecting IFN release [41]. The interaction of viral NSs with IRF7 and IRF3 and the subsequent immobilization of these transcription factors into the viral envelope is a unique strategy used by this vein virus to ensure effective evasion and suppression of host innate immunity.

NF-κB: NF-κB, an important transcription factor, has various biological functions during viral infection, such as pro-inflammatory, antiviral, and apoptotic responses. It was found that TBK1 inhibits the NF-κB signaling pathway and cytokine/chemokine induction in a kinase-activity-dependent manner and that NSs of SFTSV isolate TBK1 to prevent its inhibition of NF-κB, thereby promoting the activation of NF-κB and its target cytokine/chemokine genes [42]. another study discovered that SFTSV infection led to signifcant increases in proinflammatory cytokines and chemokines regulated by NF-κB signaling in liver epithelial cells, and the activation of NFκB signaling during infection promoted viral replication in liver epithelial cells [43].

Inflammatory factors. Interleukin-1β (IL-1β) is one of the powerful inflammatory factors, and the maturation and secretion of IL-1β is mediated by the nucleotide and oligomeric structural domain, leucine-rich repeat protein family, and nod-like receptor protein 3 (NLRP3) [44]. In macrophages, BCL2 antagonist 1 (BAX) and BAK/BCL2-related X (BAK) activation leads to the degradation of inhibitor of apoptosis (IAP) protein, which promotes caspase-8-mediated activation of IL-1β. Notably, upon BAX/BAK activation, the apoptosis enforcers caspase-3 and caspase-7 act upstream of caspase-8 and NLRP3-induced IL-1β maturation and secretion. This demonstrates the ability of innate immune cells with BAX/BAK-mediated apoptosis to generate pro-inflammatory signals [45]. In addition, Li et al. found that SFTSV infection triggers BAK upregulation and BAX activation, leading to mitochondrial DNA oxidation and subsequent cytosol release and triggering NLRP3 inflammatory vesicle activation [46]. This was also demonstrated in the study of Gao et al. The N-terminal fragments of NSs, i.e., amino acids 1 to 66, promote inflammasome complex assembly by interacting with NLRP3 [47]. Excessive NLRP3 activation leads to IL-1β secretion and ultimately to severe fever in the body. In one paper, it was reported that viral load was positively correlated with the inflammatory factors IL-6 and IP-10 and negatively correlated with RANTES in 100 SFTS patients. This is because SFTSV can cause cytokine changes; that is, there are different degrees of fluctuations in cytokine levels in patients after the onset of the disease. IL-6 and IL-8 levels in the asymptomatic infection group differed significantly between the SFTS patient group and the healthy human group [48]. This was also demonstrated in an experiment with mouse hepatocytes, where infection of hepatic epithelial cells resulted in significant increases in pro-inflammatory and chemokines such as IL-6, RANTES, IP-10, and MIP-3a [43].

**Figure 1:**
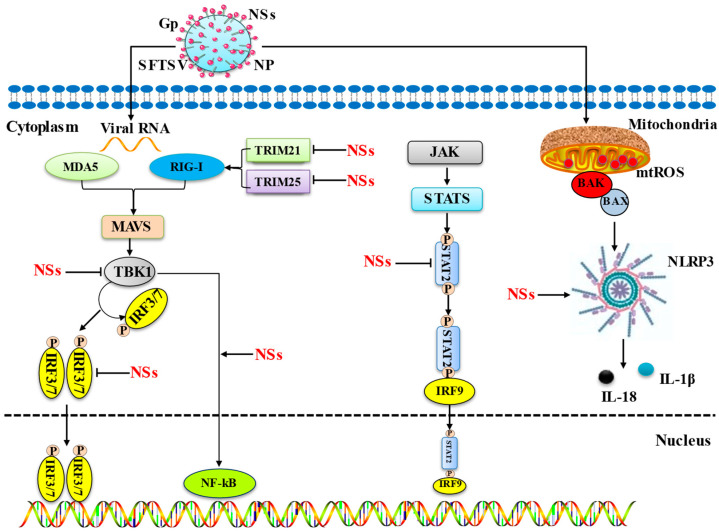
Mechanisms by which SFTS inhibits key immune signalling pathways and promotes inflammation. This diagram illustrates the main mechanism by which the host cell pathway is impacted by the NS proteins of SFTSV. Interferon release, the primary signaling mechanism for immune evasion, is inhibited by NSs in a number of ways, including the following: (1) NSs inhibit IFN production by isolating TRIM21/25, TBK1, STAT1/2, and IRF3/7 into their inclusion bodies. (2) Inhibition of the JAK/STAT signaling pathway, thereby inhibiting IFN production. The toxic NS proteins of SFTS induce inflammation through two pathways, including the following: (1) NSs produce inflammatory factors by promoting the transcription of NF-kB into the nucleus. (2) NSs trigger BAK upregulation and BAX activation through infection, leading to mitochondrial DNA oxidation and subsequent cytosol release and triggering NLRP3 inflammasome activation, which leads to inflammatory factor production.

The mechanisms by which SFTS inhibits key immune signaling pathways and promotes inflammation are summarized in Figure 1.

Autophagy and viral replication. After infection with SFTSV, cells stop at the G2/M transition, and the accumulation of cells at the G2/M transition does not affect viral adsorption and entry, but this does promote viral replication The interaction between viral NSs and CDK1 inhibits the formation of the cell cycle protein B1–CDK1 complex and nuclear import, leading to cell cycle arrest, and the expressed CDK1 loss-of-function mutants reverse the inhibitory effect of NSs on the cell cycle, thereby promoting viral replication [49]. In addition, it has been shown that SFTSV triggers a classical RB1CC1/FIP200-BECN1-ATG5-dependent autophagic flux and that the nuclear protein of SFTSV induces BECN1-dependent autophagy by disrupting the BECN1–BCL2 association. Importantly, SFTSV uses autophagy for the viral life cycle, which not only assembles in autophagosomes derived from the ERGIC and Golgi complexes, but also uses autophagic vesicles for extravasation [50].

The nuclear matrix protein nuclear scaffold attachment factor A (SAFA). Also known as heterogeneous ribonucleoprotein U, SAFA was recently identified as a novel nuclear RNA sensor that, upon recognition of viral double-stranded RNA (dsRNA), oligomerizes in the nucleus and functions as a super enhancer to promote activation of antiviral responses through interaction with chromatin remodeling complexes. SAFA recruits and promotes activation of the STING–TBK1 signaling axis against SFTSV infection, and SAFA functions as a novel cytoplasmic RNA sensor [51].

Arginine metabolic pathways. Through metabolomic analysis of two independent cohorts of SFTS patients, Li et al. found arginine deficiency in SFTS cases, suggesting that arginine metabolism by nitric oxide synthase and arginase is a key pathway for SFTSV infection and consequent death [52]. Moreover, arginine deficiency is associated with reduced intraplatelet nitric oxide (Plt-NO) concentrations, platelet activation, and thrombocytopenia.

**Figure 2:**
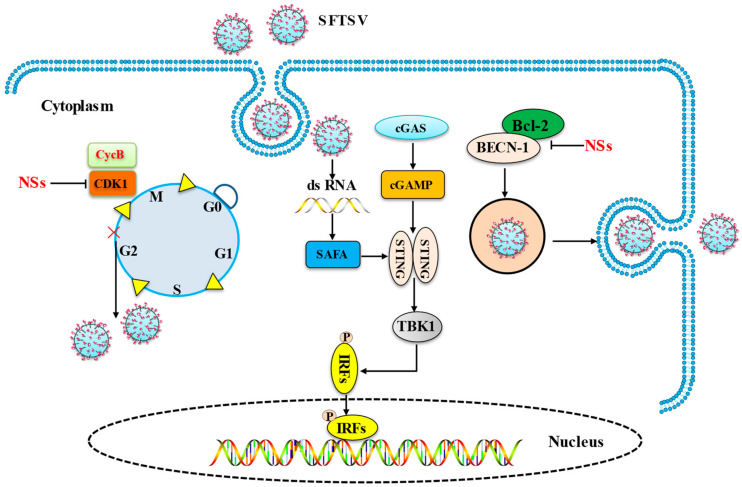
SFTSV mediates cell cycle, apoptosis, and the cGAS-STING pathway to achieve immune escape. The main mechanism by which the SFTSV influences the host cell pathway is depicted in this picture. (1) NSs inhibit host cell cycle protein CDK1 to achieve viral replication. (2) SAFA recruits and promotes activation of the STING–TBK1 signaling axis against SFTSV infection; (3) NSs induce BECN-1-dependent autophagy by breaking the BECN1–BCL2 linkage and complete virus replication.

The SFTSV mediation of cell cycle, apoptosis, and the cGAS-STING pathway to achieve immune escape is summarized in Figure 2.

## 4. Adapt Immunity

T cells: T lymphocytes are the primary immune cells that mediate cellular immune responses. Defective serologic responses to SFTSV have been found to correlate with disease morbidity and mortality, and T-cell damage leads to the disruption of antiviral immunity. Amplified and impaired antibody secretion is a hallmark of fatal SFTSV infection. Monocyte apoptosis early in infection reduces antigen presentation by dendritic cells, impedes T follicular helper cell differentiation and function, and leads to the failure of virus-specific humoral responses [53]. Yi et al. studied the dynamics of circulating regulatory T cells (Tregs) in SFTS patients at different stages. The results showed that the ratio of CD4^+^/total lymphocytes and CD4^+^CD25^+^/CD4^+^cells was significantly higher in the non-severe group than in the severe group. In contrast, the proportion of CD4^+^CD25^+^Foxp3^+^/CD4^+^CD25^+^ cells was lower in the non-severe disease group than in the severe disease group. Furthermore, during the recovery period from SFTSV infection, circulating Tregs returned to the normal range. Tregs levels correlated with various clinical parameters, which demonstrated that SFTSV infection leads to a strong response of circulating Treg in SFTS patients [54].

B cells: B cell immune responses are regulated by antigen-presenting cells and Tfhs. It has been found that, during fatal infection, SFTSV leads to an excessive inflammatory response by significantly inducing abnormal inactivation of pro-inflammatory cytokines and chemokines as well as adaptive immune responses. Furthermore, the majority of SFTSV in fatal infections is found in plasma B cells. Thus, SFTSV infection inhibits the maturation and secretion of high-affinity antibodies in plasma B cells and suppresses the production of neutralizing antibodies, leading to significant viral replication and subsequent death [55]. In addition, a defective serologic response to SFTSV has been found to correlate with disease morbidity and mortality, with a combination of B and T cell damage leading to the destruction of antiviral immunity. The serology of deceased patients was characterized by a lack of specific IgG against the viral nucleocapsid (NP) and glycoprotein (Gn) owing to the failure of B-cell class switching [56]. In addition, Suzuki et al. found that B cells that differentiate into plasma cells and macrophages in secondary lymphoid organs are the end-stage SFTSV infection targets of SFTSV infection and that the majority of SFTSV-infected cells were B cell lineage lymphocytes. In infected individuals, SFTSV-infected B cell lineage lymphocytes are widely distributed in lymphoid and non-lymphoid organs, and the human plasmacytoid lymphoma cell line PBL-1 is susceptible to SFTSV transmission and has an immunophenotype similar to that of SFTSV lethal SFTS target cells [56].

Macrophages: miR-146a and miR-146b were significantly upregulated in macrophages during SFTSV infection, driving macrophage differentiation to M2 cells by targeting STAT1. Further analysis showed that elevated miR-146b, but not miR-146a, acted on IL-10 stimulation. In addition, SFTSV increased macrophage differentiation toward M2 cells mediated by viral nonstructural proteins (NSs) induced by endogenous miR-146b. Macrophage differentiation toward M2 bias may be important for the pathogenesis of SFTS [57].

Natural killer (NK) cells: NK cells are effector cells of the innate immune system that specialize in recognizing and destroying virally infected cells during the early stages of infection. NK cells in human peripheral blood can be divided into the following five NK subsets based on the relative expression of the markers CD16 (or FcγRIIIA, the low-affinity receptor for the Fc portion of immunoglobulin G) and CD56 (an adhesion molecule that mediates isotype adhesion): CD56^bright^CD16^−^, CD56^briht^CD16^+^, CD56^dim^CD16^−^, CD56^dim^CD16^+^, and CD56^−^CD16^+^ [58]. Studies have shown that CD56^dim^CD16^+^ NK cells are associated with increased severity of SFTS, with higher levels of Ki-67 and granzyme B expression in CD56^dim^CD16^+^ and lower expression levels of NKG2A. Increased effector function of NK cells as well as CD56^dim^ NK cells was observed in the acute phase of SFTS patients [59].

**Figure 3:**
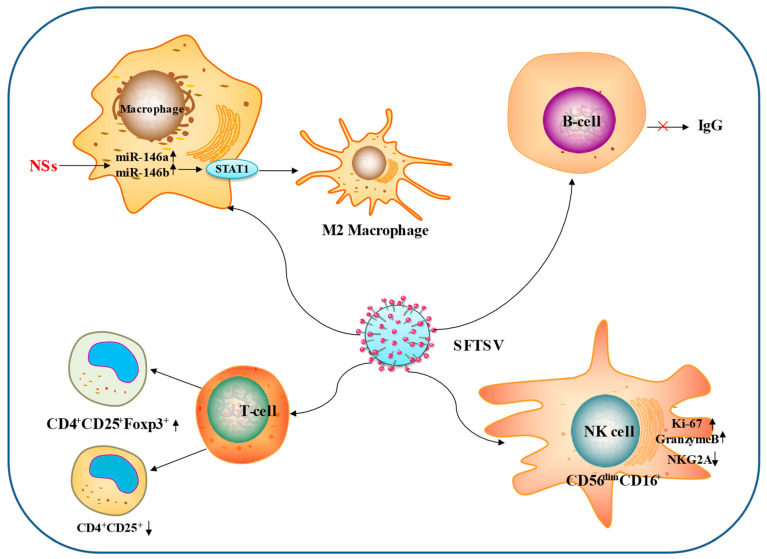
Interactions between SFTSV and immune cells. The main ways in which SFTSV influences the immune cells through adaptive immunity are depicted in this diagram. (1) SFTSV increased the differentiation of macrophages into M_2_ cells NS-mediated endogenous miR-146b. (2) SFTSV increased the expression of Ki-67 and granzyme B in CD56^dim^CD16^+^ NK cells. (3) SFTSV inhibits IgG antibody production by B cells. (4) SFTSV promotes CD4^+^CD25^+^Foxp3+ T cell production and suppresses CD4^+^CD25^+^ T cells.

The interactions between SFTSV and immune cells are summarized in Figure 3.

## 5. Study of Antiviral Drugs against SFTSV

### 5.1. Ribavilin 1-β-d-Nuclear Furanyl-1,2,4-Triazole-3 Carboxamide

Ribavirin is a synthetic, broad-spectrum antiretroviral drug that also has anti-tumour activity. It is active against respiratory and enteroviruses, influenza viruses, herpes viruses, human immunodeficiency virus, lassa virus, and hepatitis C virus. The mechanism of antiviral action of ribavirin is not yet clear. Studies in the relevant literature suggest that an important aspect of ribavirin’s antiviral activity may arise from the ability to act simultaneously through multiple mechanisms. Ribavirin may act at multiple steps in the viral life cycle: (1) inhibition of translocation by reducing the cellular GTP pool or by incorporation as a translocation-inhibiting capsid analogue; (2) inhibition of genomic or transcript capping by inhibition of GTP synthesis or direct competition; (3) direct inhibition of RNA synthesis or reduction in GTP synthesis by binding to the active site; (4) blurring incorporation into RNA and resulting in viral response; (5) strengthening antiviral immune responses to stop transmission and morbidity, increasing mutation and the generation of non-viable genomes, or hazy integration into RNA [60]. However, some scholars have studied the clinical efficacy of ribavirin, with their results showing that ribavirin did not have much effect on the treatment of SFTSV virus infection [61], so it remains to be examined whether ribavirin can effectively treat SFTSV virus infection.

### 5.2. Favipiravir T-705 (6-Fluoro-3-Hydroxy-2-Pyrazine Carboxamide)

Favipiravir is structurally a pyrazine derivative and a novel antiviral drug [62] that binds to and inhibits the RNA-dependent RNA polymerase (RdRp) in RNA viruses and finally prevents the transcription and replication of viral RNA, thereby exerting antiviral effects. Therefore, it has shown broad antiviral activity against a variety of RNA viruses [63]. These include influenza virus, sandy virus, Bunyavirus, West Nile virus, and foot-and-mouth disease virus [64]. Rui Song et al. [65] studied two patients with varying degrees of SFTSV infection and found that both patients were cured by treatment with favipiravir. This study also suggests that favipiravir has potential anti-SFTSV viral activity. In addition, several studies have been reported on the effectiveness of famipiravir in reducing morbidity and mortality in SFTS patients [66,67], but the side effects caused by the use of famipiravir remain to be studied.

### 5.3. Hexachlorophenol (Hexachlorophene)

Yuan et al. used ELISA to do an initial screening of a library of 1528 medicinal compounds licensed by the FDA, and they verified it using assays for viral reduction. Hexachlorophenol was determined to have the best activity. According to mechanistic research, hexachlorophen prevents SFTSV from entering host cells by interfering with cell membrane fusion. Hexachlorophen’s highly stable binding to the hydrophobic pocket between the structural domains of SFTSV Gc glycoproteins I and III was predicted by molecular docking research. Hexachlorophen’s newly discovered antiviral properties and mechanisms will enable it to operate as a lead molecule for the creation of further inhibitors with increased anti-SFTSV activity and reduced toxicity [68].

### 5.4. Ion Channel Blockers

Calcium channels: Calcium channel blockers (CCBs) are drugs that block the entry of calcium ions into cells via calcium channels in the cell membrane. Calcium channel blockers are widely used clinically for the treatment of hypertension and are particularly effective in elderly patients. They are also used to regulate heart rate, prevent cerebral vasospasm, and reduce pain caused by angina pectoris [69]. By screening the FDA-approved drug library, Hao Li et al. [69] found that benidipine hydrochloride and nifedipine had in vitro inhibitory effects on SFTSV virus. Further experiments in mice yielded that Benidipine hydrochloride could combat SFTSV virus infection by inhibiting viral internalization and genome replication. Nifedipine is mainly used clinically as an anti-hypertensive drug, but also has some anti-SFTSV viral activity. CCBs are considered to be potentially effective therapies for the treatment of acute and severe SFTSV infections and for reducing SFTSV-related morbidity and mortality.

Potassium channels: Drugs that act on K+ channels are mainly class III antiarrhythmic agents in clinical practice and exert their antiarrhythmic effects by prolonging the process of cardiomyocyte action potential and prolonging repolarisation. In this literature, it has been found that Bunyavirus infection may be associated with the activation of K+ channels. Moreover, K+ is thought to be a key regulator of its infection process [70]. In addition, potassium channels can be activated for a short period of time during the early stages of viral infection [71] to facilitate viral infection of the organism.

### 5.5. IFN-γ

IFN-γ has cytostatic, apoptosis-promoting, and immune-inducing effects and plays an important role in the recognition and elimination of transformed cells [72], and is the only member of the type I interferon family. It also induces direct antimicrobial activity by stimulating macrophages and dendritic cells through modulation of antigen processing and delivery pathways. Mechanistic studies have shown that IFN-γ can act as a direct stimulator of the expression of certain potential antiviral proteins via STAT1 signaling, which is a direct mechanism of resistance to SFTSV virus infection, leading to the conclusion that it may also play an important role in viral infections [73].

### 5.6. 2′-Fluoro-2′-Deoxycytidine

2′-Fluoro-2′-deoxycytidine is a nucleoside analogue that is a potent inhibitor of Congo haemorrhagic fever virus (CCHFV) replication. 2′-Deoxy-2′-fluorocytidine acts synergistically with T705 to enhance the antiviral efficacy of both compounds against CCHFV replication. It has previously been shown to have some antiviral effects against HIV as well as herpes viruses [74]. It has also been found that 2′-FdC has a broad inhibitory effect against certain Bunyaviruses in in vitro cell culture, with better antiviral effects compared with ribavirin [75]. Moreover, 2′-Fluoro-2′-deoxycytidine has shown good therapeutic promise in vivo against certain venereal viral infections [75].

### 5.7. Caffeic Acid (CA)

An organic acid associated with coffee as well as a degradation product of chlorogenic acid [76], which is present in large quantities in coffee beans. It is the effect of caffeic acid that gives coffee its deep aroma, colour, and bitter taste. CA has various beneficial biological effects, including inhibition of metastasis and proliferation of cancer cells [77,78,79] and antiviral [80,81] effects. Motohiko Ogawa et al. [82] concluded, both by adding SFTSV to CA-containing media and by pre-incubating SFTSV with CA, that pre-incubation greatly reduced viral cell attachment. CA treatment of SFTSV-infected cells was also able to inhibit viral infection to some extent. This leads to the conclusion that CA may inhibit virus transmission by acting directly on the virus rather than on the infected cells. They also demonstrated that CA is a potential anti-SFTSV drug for the prevention and treatment of SFTSV [82], which provides a valuable reference for the subsequent development of anti-SFTSV drugs.

### 5.8. Amodiaquine (Amodiaquine)

Amodiaquine is a new compound used clinically to treat acute attacks of malaria and to control the symptoms of malaria. It is also used in the treatment of amebiasis of the liver, Schistosoma haematobium, Schistosoma mansoni, and connective tissue disease. It is also used to treat photosensitivity disorders such as solar erythema. Amodiaquine has also been found to have antiviral activity against Ebola virus [83], dengue virus, and Zika virus [84], but its mechanism of inhibition against malaria and the above-mentioned viruses remains to be confirmed. In order to find novel compounds with anti-SFTSV activity, a safe and rapid assay system was developed by Baba et al. [85]. The anti-SFTSV drugs were screened by infecting SFTSV cells with different dilutions of the drug and further culturing them under these conditions, followed by the use of kits and SFTSV-specific TaqMan primers/probes and a real-time RT-PCR assay of intracellular SFTSV RNA. Amodiaquine and its derivatives were found to inhibit SFTSV replication in vitro by this systematic assay.

### 5.9. Immunotherapy

(1) Glucocorticoids

Glucocorticoids have a wide range of therapeutic effects and are used to treat severe acute infections or inflammation, asthma, and autoimmune diseases, as well as to prevent the sequelae of inflammation, among other things. Severe SFTS infections may cause a cytokine storm [86] in patients, resulting in a severe inflammatory response, leading to death. Based on the pathogenic mechanism of SFTS, glucocorticoids can be used to counteract the inflammatory response of the cytokine storm and relieve the clinical symptoms of the patient. Meanwhile, scholars from other countries have reported several cases of steroids combined with other drugs successfully treating SFTSV virus infection and even SFTSV virus infection combined with encephalopathy [87,88]. They used pulse therapy for SFTSV virus infection combined with encephalopathy, while noting that short-term glucocorticoid therapy may be beneficial in treating encephalopathy in the early stages of SFTSV infection. Although glucocorticoid therapy can rapidly relieve patients’ clinical symptoms, it can itself aggravate the chances of infection and lead to secondary infections. There are also no more adequate literature reports demonstrating that glucocorticoids can be used to treat SFTSV virus infection, and subsequent studies of this class of drugs are needed to explain whether they are effective in treating SFTSV virus infection.

(2) Plasma exchange

TPE is primarily achieved by removing pathological inflammatory mediators, including autoantibodies, complement components, and cytokines, and is commonly used in a number of autoimmune diseases. This therapy also targets the cytokine burst caused by SFTSV viral infection and suppresses the inflammatory response it causes [89]. By summarizing the observations of patients with SFTSV viral infection who used TPE therapy at a tertiary care hospital, Jeong Rae Yoo et al. [89] concluded that those SFTS patients who received TPE therapy showed clinical, laboratory, and virological parameters. The results of the study also showed that TPE is a useful tool in the treatment of SFTS. The results also suggest that TPE can be used as a salvage treatment for patients with rapidly progressive SFTS. At the same time, the use of plasma exchange in the treatment of SFTSV infection is only at the experimental stage and further clinical trials are needed to determine whether this therapy is safe, effective, and able to be widely used in the treatment of SFTSV infection.

(3) New therapeutic approaches

Monoclonal antibodies were first produced using mouse proteins, which were not well tolerated in humans owing to species differences, thus stimulating the development of monoclonal antibody technology and eventually monoclonal antibodies suitable for humans [90]. As a new therapeutic approach, monoclonal antibodies are considered to be promising for the treatment of SFTS [91]. Furthermore, Shimade et al. found that post-exposure prophylaxis using human antiserum from SFTS patients had a significant therapeutic effect on SFTSV infection in an in vivo mouse model, a result that strongly suggests that antiserum treatment may be clinically useful for post-exposure prophylaxis of SFTSV infection [92].

### 5.10. Vaccine

Vaccines are our most powerful biological weapon against the prevalence and occurrence of infectious diseases. In the face of SFTSV virus infections, the development of targeted vaccine products is also a matter of urgency. Some scholars are currently working on the design of vaccines against SFTSV based on the highly attenuated poxvirus strain LC16m8 [93], with a view to providing direction for subsequent vaccine development. They applied the highly attenuated but still immunogenic poxvirus strain LC 16 m8 (m8) as a recombinant vaccine against SFTS. Recombinant m8 was generated expressing SFTSV nucleoprotein (m8-N), envelope glycoprotein precursor (m8-GPC), and both N and GPC (m8-N+GPC) in infected cells, and it was also confirmed that both m8-GPC- and m8-N+GPC-infected cells produced SFTSV-like particles (VLP) in vitro. It was demonstrated through a series of experiments that the obtained recombinant m8- GPC and m8-N+ GPC were considered as promising vaccine candidates for SFTS and provide a reference for subsequent vaccine development [93]. Jeong-Eun Kwak et al. investigated the SFTSV DNA vaccine for ferrets and examined its protective effect in lethal SFTSV virus infection [94]. Through lethal infection experiments in aged ferrets, vaccinated in a lethal infection test in older ferrets, vaccinated ferrets showed good antiviral effects and did not show clinical signs of infection, suggesting that the vaccine could be useful in the development of an anti-SFTSV vaccine.

## 6. Chinese Medicinal Ingredients against SFTSV Virus

Li, S. et al. [95] obtained three compounds, Notoginsenoside Ft1, Punicalin, and Tosendanin, with high anti-SFTSV activity through high-throughput screening of a library of natural extracts of compounds with anti-SFTSV infection activity. Among them, Tosendanin showed the highest inhibitory capacity. Tosendanin is a type I voltage-dependent calcium channel agonist, which may be involved in the regulation of intracellular calcium homeostasis. Mechanistic studies have shown that Tosendanin can inhibit SFTSV infection during the internalization phase [95]. The antiviral effect of Tosendanin on SFTSV was further demonstrated in a mouse infection model, where Tosendanin treatment resulted in a significant reduction in viral load and histopathological changes in mice. The study also revealed that the antiviral activity of Tosendanin could be further extended to another bunyavirus and the emerging SARS-CoV-2. This will facilitate the discovery and development of anti-bunyavirus drugs.

## 7. Conclusions and Future Perspectives

In the decades since the discovery of SFTSV in China, a great deal of research has been collected on viral vectors, virology, and the way the virus invades the organism, as well as on drugs that target the virus. However, a considerable number of questions remain to be answered, such as the existence of SFTSV transmission from other mammals to humans, the transmission cycle of SFTSV, the complex immune response to the host, and so on. SFTSV causes mortality mainly by causing leukocytopenia and multi-organ failure and by causing severe fever in the body, leading to immune system failure, yet no effective treatment has been established. The non-structural domain NS proteins of SFTSV act as its main virulence protein and, by inhibiting host cell IFN synthesis and producing a large number of inflammatory factors, SFTSV disables the host cell immune system. It is likely that SFTSV has been circulating in China and the world for a considerable period of time, but has not been detected during this time, and it is likely that more strains of the virus will evolve over time. More information on the virus will certainly be gathered in the coming years, but the full extent of the human disease risk posed by SFTSV has not yet been fully determined. The death rate from SFTS is gradually decreasing as global medical care improves, but there is no fully effective vaccine or drug that can cure it, so an effective vaccine or drug has yet to be developed. Scholars and clinicians should actively share their experiences and continue to improve the information and treatment of the virus in order to combat the disease.

## Data Availability

The data that support the findings of this study are available from the corresponding author upon reasonable request.
